# Painless Capillary Blood Collection: A Rapid Evaluation of the Onflow Device

**DOI:** 10.3390/diagnostics13101754

**Published:** 2023-05-16

**Authors:** Lara Dominique Noble, Caitlin Dixon, Alison Moran, Charlotte Trottet, Mohammed Majam, Shameema Ismail, Vanessa Tiyamike Msolomba, Kegomoditswe Mathobela, Arthur Queval, Jaya George, Lesley Erica Scott, Wendy Susan Stevens

**Affiliations:** 1 WITS Diagnostic Innovation Hub, University of the Witwatersrand, Johannesburg 2000, South Africalesley.scott@wits.ac.za (L.E.S.);; 2 Loop Medical SA, 1015 Lausanne, Vaud, Switzerland; 3 Ezintsha, a Sub-Division of Wits Reproductive Health and HIV Institute, University of the Witwatersrand, Johannesburg 2000, South Africa; 4 National Priority Programmes, National Health Laboratory Service, Johannesburg 2000, South Africa

**Keywords:** capillary blood collection, upper arm collection device, pain-free, venous-comparable blood specimen, serum chemistry, onflow serum gel

## Abstract

Blood-based diagnostics are critical for many medical decisions, but mostly rely on venepuncture, which can be inconvenient and painful. The Onflow Serum Gel (Loop Medical SA, Vaud, Lausanne, Switzerland) is a novel blood collection device that utilises needle-free technology to collect capillary blood. In this pilot study, 100 healthy participants were enrolled and provided two Onflow collected specimens and one venous blood specimen. Five chemistry analytes (AST, ALT, LDH, potassium, creatinine) and haemolysis were measured per specimen, and laboratory analyte results were compared. Onflow was found to be more acceptable than venepuncture with lower pain ratings, and 96.5% of participants would use the Onflow method again. All phlebotomists (100%) found Onflow intuitive and user-friendly, with ~1 mL of Onflow blood successfully collected from 99% of participants in <12 min (mean: 6 min, 40 s) and 91% collected on the first attempt. ALT and AST analytes showed no difference in performance, while creatinine generated a negative bias (−5.6 µmol/L), and increased variability was noted with potassium (3.6%CV) and LDH (6.7%CV), although none were clinically relevant. These differences may be due to 35% of Onflow collected specimens having “mild” haemolysis. Onflow is a promising alternative blood collection device that should now be evaluated in participants with expected abnormal chemistries and as an option for self-collection.

## 1. Introduction

Venous blood collection requires skilled healthcare practitioners trained in phlebotomy procedures, which is often unavailable in resource-limited settings (RLS). There is also an increased preference for patient-centric sampling and methods that are not only “pain-free” but also maintain specimen quality [1]. Some of these alternative technologies draw only small blood volumes (less than 30 microlitres (µL)), which are not suitable for multi-analyte and automated testing. Four commercial products, however, are able to obtain 400–600 µL capillary, whole blood for testing. The BD Microtainers (Becton Dickinson and Company, Franklin Lakes, New Jersey, United States) are small tubes designed for capillary blood collection (approximately 600 µL) from a finger stick specimen [1,2,3,4]. The Haiim Vacuum Assisted Blood Collection device (Winnoz, New Taipei City, Taiwan) uses a lancet and small vacuum device to collect up to 500 µL whole blood from a finger stick but does require a trained HCP (health care practitioner) to oversee the collection [1,5]. The TAP^®^ II (up to 350 µL) and TAP^®^ micro (up to 600 µL) devices (YourBio Health, Medford, MA, USA) use a microneedle array to collect capillary blood, usually from the upper arm, using a vacuum [6]. The Tasso+ and Tasso^®^ micro devices (Tasso Inc., Seattle, WA, USA) collect 400–600 µL (tube-dependent) capillary whole blood from the upper arm using a microneedle and a vacuum [7]. The widely evaluated Tasso-SST device collects ~300 µL whole blood [1,8,9,10,11] into a tube that contains a clot activator and separation gel but is no longer available [12]. To date, these devices have been assessed for clinical chemistry [3,4,13,14,15], haematology [2], and serology [8,9,10,11,16,17,18], with overall good analytical performance and participant acceptance. It must be noted, however, that most automated analysers require a dead volume (200–300 µL) of specimen in addition to the volume required for analyte testing. In addition, the source specimen is frequently plasma or serum, which makes up ~55% of the whole blood specimen [19], meaning that the volume of blood obtained is not the volume of specimen available for testing. None of the existing studies had difficulties with the volume of specimens obtained for their analyses, although the lack of specimens for repeat testing was raised [3,13]. Specimen self-collection using these devices has also been reported [3,4,17].

**Figure 1:**
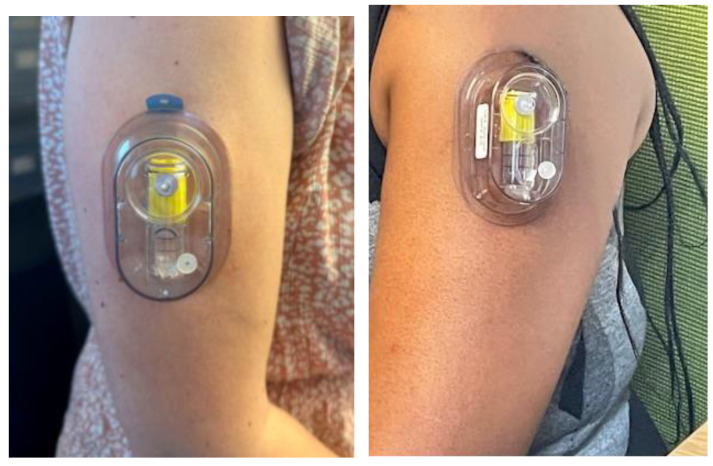
Examples of the Onflow device placed on the upper arm.

Loop Medical SA (Vaud, Lausanne, Switzerland) has developed a novel capillary blood collection device called the Onflow (www.loop-medical.com, accessed 11 May 2023) and Figure 1), which is a single-use blood collection device that accesses capillary blood between the skin surface and the nerve layer, usually from the upper arm, and is expected to be less painful than both venepuncture and finger stick blood collection. The device collects approximately one millilitre (mL) of capillary whole blood into a small phlebotomy tube in 5–10 min, without compromising patient safety or specimen quality. The key advantage of the Onflow device is that it minimises the need for trained phlebotomists as it can be applied by non-specialised HCP and potentially by patients themselves for specimen self-collection. This study describes a pilot evaluation of the Onflow device to measure the quality of the blood drawn through the analytical performance, and ease of use and acceptance of the Onflow compared to venepuncture. 

## 2. Materials and Methods

### 2.1. Clinical Study Processes

This study was performed among HCP and staff at Ezintsha Clinic in Johannesburg, South Africa. The study was approved by the University of the Witwatersrand Human Research Ethics Committee (WITS HREC: M210731) and the South African Health Products Regulatory Authority (SAHPRA: DMMH008_V1.3). A total of 100 HCP were invited to participate, with exclusion criteria being persons presenting with oedema or open wounds, abnormal skin integrity on the upper arm, subcutaneous implants in the upper arm, known stainless steel allergies, haemophilia and those receiving anticoagulant therapy. Two Onflow specimens (one per arm) and one venous blood specimen were collected by trained phlebotomists from each enrolled participant. Participants were asked to warm the capillary blood collection site by rubbing their upper arm prior to site sterilisation and application of the Onflow device. The Onflow device is equipped with its own internal serum separation tube (Serum Gel) into which the sample was directly deposited during collection. All venous blood samples were collected into 3.5 mL serum separator tubes (BD SST™) (SST; Becton Dickinson and Company, Franklin Lakes, New Jersey, United States). The specimen collection order was randomised, and specimens were collected sequentially. Post-blood collection, the collection site was examined according to dermal response scoring ([20] and Table 1) and the level of pain and acceptability experienced was rated by the participant. The study phlebotomists also rated the condition of devices and their ease of use and answered questions outlined in Table 1. 

### 2.2. Laboratory Specimen Processing 

Once blood was collected by the Onflow devices, the tubes were removed from the devices and stored upright along with the venous blood SSTs at ambient temperature (~23 °C) for a maximum of 2 h before transport to the testing laboratory (clinical laboratory platform of the WITS Diagnostic Innovation Hub (WDIH, Johannesburg, South Africa). The transport from the collection site to the testing laboratory is approximately a 5-min drive. At the laboratory, the Onflow blood tubes were weighed using an OHAUS^®^ Navigator™ scale (OHAUS Corporation, Parsippany, NJ, USA), and three decimal points were recorded. The approximate blood volume was obtained by subtracting the mean tube weight (empty) from the total weight and dividing it by the density of blood (1.06 g/mL [21]). The specimens were centrifuged at 5000× *g* for 2 min to separate the serum from the blood cells. The haemoglobin concentration (g/dL) was measured using 10 µL of serum tested on the HemoCue^®^ Plasma/Low Hb System (HemoCue AB, Ӓngelholm, Sweden). The absolute result was converted to g/L, which was used as a measure of haemolysis according to conventional haemolysis classifications [22]. Briefly, haemolysis was defined as not haemolysed (free haemoglobin ≤0.25 g/L) or as having insignificant (free haemoglobin >0.25 g/L and ≤0.5 g/L), mild (free haemoglobin >0.5 g/L and ≤3.0 g/L), moderate (free haemoglobin >3.0 g/L and ≤5.0 g/L) or gross (free haemoglobin >5.0 g/L) haemolysis [22]. The remaining serum in the Onflow tube was transferred into clear Polystyrene, round-bottom test tubes (JPlast Plastic Products, Roodepoort, Gauteng, South Africa) and five analytes were tested on the cobas c 501 chemistry analyser (Roche Molecular Systems, Pleasanton, CA, USA): aspartate transaminase (AST), alanine transaminase (ALT), potassium, creatinine and lactate dehydrogenase (LDH). Residual specimens were stored at 4 °C. The ALT analyte was measured from refrigerated venous and Onflow specimens (within seven days of being stored) for the first 49 specimens. 

### 2.3. Data Analysis

Clinical and laboratory data were captured into Smart-Trial electronic data capture (Indianapolis, IN, USA) software. The combined data were exported as a Microsoft™ Excel (Microsoft Corporation, Redmond, WA, USA) file for subsequent analyses. Data were analysed using MedCalc (Ostend, West-Vlaanderen, Belgium) and R (R Foundation for Statistical Computing, Vienna, Austria). The Onflow specimen results were individually compared to the venous specimen results for each analyte, with the venous specimen result considered the reference. The mean absolute values, Bland–Altman mean bias [23] with 95 per cent confidence interval (95%CI), and percentage (%) similarity [24] with percentage standard deviation (%SD) and percentage coefficient of variation (%CV) were reported. The concordance correlation coefficient (P_c_) was calculated with a measure of precision (Pearson, ρ) and a measure of accuracy (bias correction factor, c_b_) [25]. Overall agreement was considered poor for P_c_ < 0.9, moderate for P_c_ = 0.9–0.95, substantial for P_c_ = 0.95–0.99 and almost perfect for P_c_ > 0.99 [26]. Outliers were considered those falling outside the allowable difference from the reference (±15% for AST, ALT and LDH, ±10% for creatinine and ±0.3 mmol/L for potassium [27]). Split-violin plots (using the “introdataviz” package in R) were used for visualisation [28]. 

## 3. Results

### 3.1. Participant and Blood Draw Characteristics

The study participant demographics and specimen collection data are outlined in Table 2 and Table 3, respectively. In brief, 92% of the participants were Black African, 54% participants were female, and the overall mean participant age was 35 years. All participants had normal skin appearance before the Onflow blood draws, and only five participants (5/99) showed slight redness post blood collection. While the arm used for venous collection was evenly distributed (48.5% left arm, 51.5% right arm), the left arm was primarily used for the first Onflow device (85%) which is assumed to be a factor of clinical room set-up. All venous blood specimens were successfully collected (99% on first draw), Onflow 1 (the first draw performed with the Onflow) specimen collection was successful in 97% of attempts (91% on first attempt) with 3% collected with reduced blood volume, and all Onflow 2 (the second draw performed with the Onflow) specimens were successfully collected (92% on first attempt). The mean and median collection times for the two Onflow devices was similar (less than seven minutes), with a minimum time of 2 min and 5 s and a maximum time of 12 min and 47 s recorded.

### 3.2. Laboratory Analyte Method Comparison 

**Figure 2:**
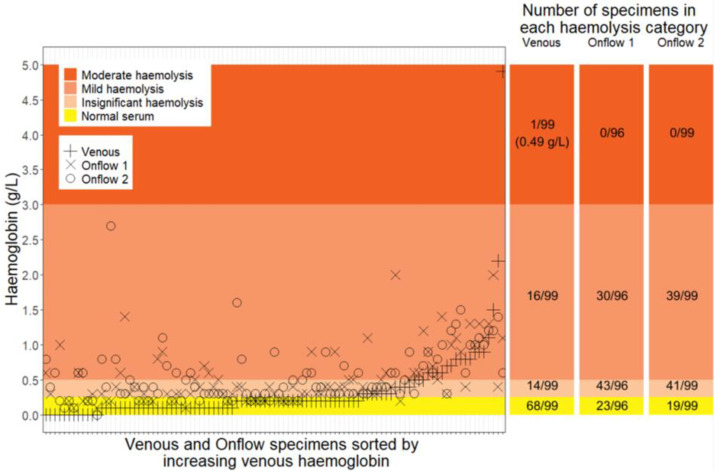
Measure of haemolysis (haemoglobin (g/L)) for each specimen collected by venepuncture and Onflow. Specimen results are sorted by increasing venous haemoglobin on the horizontal axis. The shaded areas represent the haemolysis classification [22], which are also tabulated alongside the graph. g/L: grams per litre.

The specimens successfully collected with adequate blood volume (n = 294) showed 83% (82/99) had no or insignificant haemolysis when collected by venepuncture, compared to 65% (126/195) collected by the Onflow devices as visualised in Figure 2. The Onflow collection method showed a trend of causing mild haemolysis in 35% (69/195) specimens. One specimen collected by venepuncture reported moderate haemolysis. As no moderate or gross haemolysis was observed for the Onflow devices, all specimens would have been accepted for testing based on observed haemolysis.

**Figure 3:**
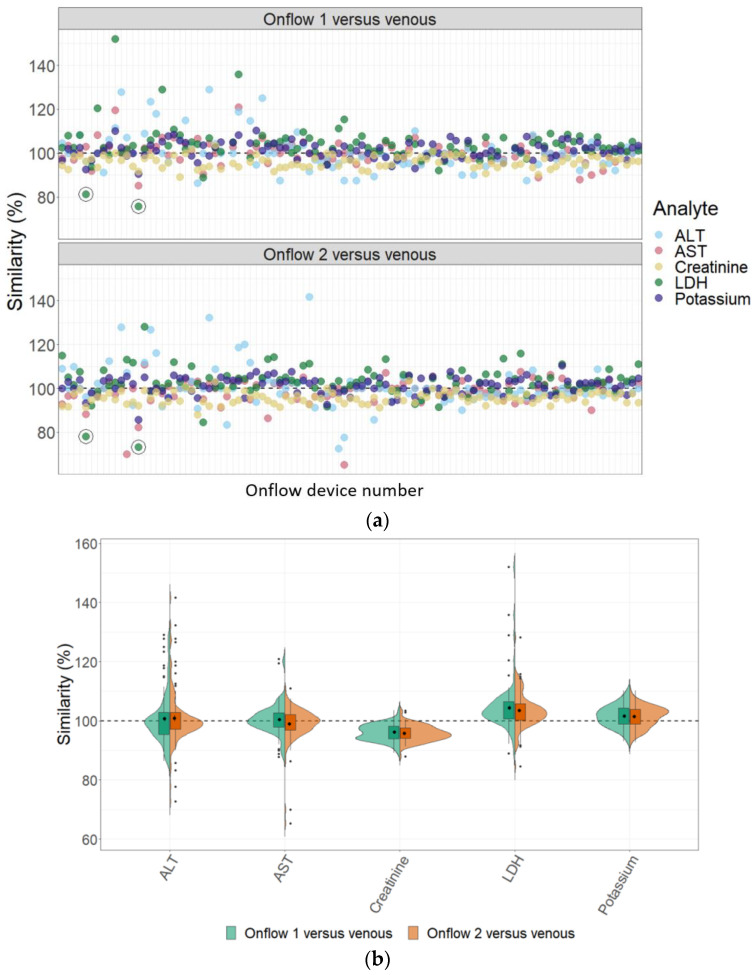
Percentage similarity analysis. (**a**) Scatter plots for Onflow 1 and Onflow 2 device test results compared to venous blood test results for all analytes from each specimen listed in sequence. Black circles highlight two results for LDH which would be clinically relevant, but both had the presence of haemolysis on venous blood. (**b**) Split-violin plots representing the percentage similarity for each Onflow device result compared to the venous blood result across the five analytes. The dotted line marks the 100% similarity reference line. %: percentage; ALT: alanine transaminase; AST: aspartate transaminase; LDH: lactate dehydrogenase.

A total of 1467 (of 1470) laboratory test results were reported, with three ALT results from two specimens that could not be generated due to insufficient volume. The percentage similarity scatter plots (Figure 3a) further highlighted two results (#5 and #14) that reported lower LDH values by Onflow compared to venous that would have been considered clinically relevant, which was linked to increased haemolysis of the venous specimens (“mild” and “moderate” haemolysis, respectively). These results were therefore removed from further method comparison of all analytes, and the total number of specimens available for statistical analysis was 1452 (98.8%). The percentage similarity, visualised by split violin plots (Figure 3b), shows on average similar test results between the two Onflow devices for all analytes. The statistical analysis was thus performed on combined Onflow results compared to the venous results. Overall, the Onflow capillary blood results were lower for creatinine and higher for LDH and potassium when compared to the venous blood results. The percentage similarity scatter plots showed less scatter beyond specimen #35 for Onflow device 1 and specimen #49 for Onflow device 2, and there is evidence of more scatter among the ALT analyte overall, which may be due to the Onflow ALT specimens #1 to #49 being stored for seven days at 4 °C prior to testing.

Table 4 summarises the method comparison outputs for the concordance correlation, Bland–Altman and percentage similarity method comparisons and includes concordance correlation plots and Bland–Altman difference plots. The mean and data range for each analyte is also listed, and it is worth noting that based on the ALT, AST, creatinine and potassium analyte ranges, specimens were received from participants reporting values in the “normal” range. The LDH results showed some elevated readings beyond the “normal reference ranges” across both venous and Onflow results. The AST and ALT analytes from Onflow collected blood substantially agreed with venous collected blood analytes. Both showed good accuracy and precision and acceptable variability. There were potentially two outliers, which were not considered clinically relevant, and which also showed greater variability in LDH measurement compared to the reference. The Onflow creatinine showed moderate agreement with venous blood creatinine, but with good precision (low %SD and ρ > 0.9). There was a definite bias (96% mean similarity and −5.6 µmol/L mean difference), but these differences were not clinically relevant (acceptable variance [27]). Although the Onflow LDH showed poor overall agreement with venous LDH, there was good accuracy (104% mean similarity and 15 U/L mean difference). The increased variance was attributed to four outliers, all of which had mild haemolysis, and which were also specimens collected within the first 30 specimens (as similarly noted with specimens #5 and #14 mentioned above). This may indicate unfamiliarity by the HCP with the use of the Onflow device. The potassium analyte comparison between Onflow and venous blood also reported poor agreement, but this was not considered clinically relevant and was due to an increased variability (reduced precision, ρ = 0.65 and percentage similarity CV = 3.6%) and one potential outlier. There was overall good accuracy (102% mean percentage similarity and 0.1 mmol/L mean difference) between Onflow and venous blood potassium analytes. 

**Figure 4:**
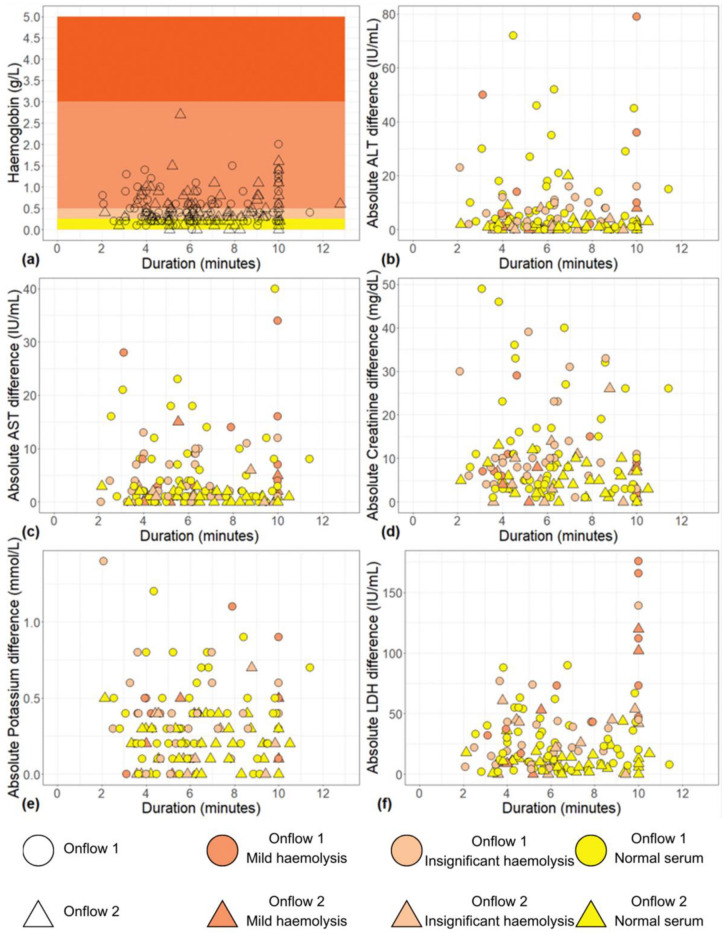
Scatter plots showing (**a**) Onflow specimen haemolysis by Onflow specimen collection time and absolute bias for the Onflow results compared to venous results for all analytes srted by specimen collection time and stratified by haemolysis ((**b**) ALT, (**c**) AST, (**d**) Creatinine, (**e**) potassium, (**f**) LDH). ALT: alanine transaminase; AST: aspartate transaminase; LDH: lactate dehydrogenase; µmol/L: micromoles per litre; mmol/L: millimoles per litre; n: number; U/L: units per litre.

To investigate any potential impact of Onflow specimen collection time on analyte results due to haemolysis, absolute bias between Onflow and venous analyte results was visualised in scatter plots (see Figure 4) according to collection time and haemolysis. Haemolysis (insignificant and mild) was observed across all specimen collection times (02.05 to 12.47), with random scatter observed for ALT, AST, creatinine and potassium analytes. Increased bias was observed among six Onflow LDH results with a collection time of ten minutes, of which five showed mild haemolysis, indicating the potential impact of haemolysis due to increased collection time on this analyte.

**Figure 5:**
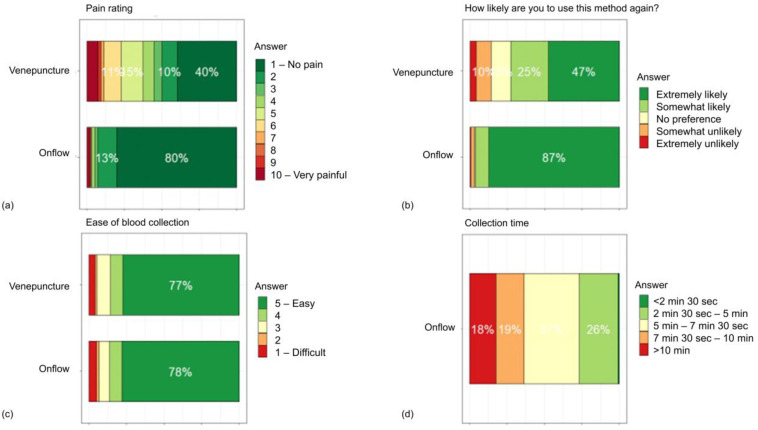
Usability and acceptability of the Onflow device compared to venepuncture (**a**) Participant pain rating for venepuncture and onflow, (**b**) Participant likelihood of using venepuncture and Onflow again, (**c**) Participant-perceived ease-of-use, and (**d**) Time taken for Onflow specimen collection.

The usability and acceptability of the Onflow devices are presented as heat bar charts in Figure 5 and clearly highlight that Onflow was more acceptable than venepuncture with lower pain ratings and that it was more likely that participants (96.5%) would use the Onflow method again. Overall, 14.1% of participants would prefer not to use venepuncture again compared to 3.0% for the Onflow device. All HCP found the Onflow device intuitive and easy to use. The devices performed as expected, with 100% described as having an intact sterile barrier, shielded blade and a tube in good condition, 100% were heard to activate, 99.5% could be activated with one hand and 99.5% of devices were closed easily. Selected HCP and participant comments are shown in Box 1.

Box 1 **User experience feedback.**

 Participants remarked that although venepuncture is quick, it is painful.

*
“Quicker but slightly painful than the loop [sic]”
*

*
“Quick but painful”
*

 Furthermore, participants expressed that they disliked venepuncture due to their fear of needles.

*
“I have a fear of needles so I do not like it when they take samples”
*

*
“Blood draw took longer but less daunting as there are no needles”
*

 The participants liked the Onflow device, with most commenting that it was pain-free and less intimidating than a needle.

*
“The procedure was easy and painless.”
*

*
“User friendly, no pain.”
*

*
“Very good to use.”*

*
“The device looks user friendly, I was not scared when she took the sample.”
*

*
“This was basically painless although takes longer than venepuncture. A much more pleasant experience mentally.”
*

 The HCP also liked the Onflow device as an alternative to venepuncture.

*
“Took longer, but no pain, good for people with difficult veins”
*

*
“It’s slow but user-friendly for children.”
*

*
“Innovative and interesting.”
*

 However, a number of participants and HCP commented that specimen collection with the Onflow device was slow.

*
“It takes too long.”
*

*
“Loop (sic) took long to draw blood”
*

*
“It is not painful, but it takes a lot of time.”
*

 Some participants also found the Onflow device uncomfortable, particularly with slow blood draws.

*
“Took quite long, did not like the feeling of it on skin—itchy & irritable.”
*

*
“The position of blood collection is a bit uncomfortable.”
*

*
“The draw took a full 10 minutes. The vacuum was a bit uncomfortable.”
*

## 4. Discussion

This is the first study, to our knowledge, reporting on the performance of the Onflow painless blood collection device. The participants were all employed in the health care field, ranging from lay HCP to nurses to project managers, and the majority of the participants were dark skin toned individuals, representative of the African region. The Onflow blood collection devices were well received by HCP applying the devices and by participants, and most preferred the Onflow method to venepuncture. This aligns with the patient feedback on the Tasso devices [4,13] and finger stick Microtainer collection [3]. Only slight redness was noted after the Onflow device use in 5/99 participants. All Onflow devices were intact and worked as per the manufacturer’s claims, with the mean collection time for 198 devices less than 7 min, although a second Onflow device was used in approximately 9 per cent of blood draws. Analyte test values that could not be obtained in our study were due to low specimen volume (including one venous specimen), rather than haemolysis. Nonetheless, an overall advantage of the Onflow device is the larger volume capacity (approximately 1 mL), and overall fewer specimens were rejected (3%), compared to 33% in a recent study with other devices [13]. It is noted, however, that in this evaluation, HCP applied the device to the participant’s arms and specimen collection success may vary when the participant applies the device (self-collection).

One notable difference between the Onflow and venepuncture collected specimens was the trend for more Onflow specimens to have mild haemolysis. Although this will not usually affect results [22], analytes should be assessed for their intended use on an individual basis ahead of implementation. In this study, it was also noted that increased Onflow specimen collection time had some impact on the measurement of the LDH analyte, while the other analytes were less affected. Future studies may use the LDH analyte to their advantage in closely monitoring the impact of increased specimen collection time on device performance. The creatinine measurements did have a negative bias, and potassium measurements showed decreased precision, but neither of these is considered to be clinically relevant and it has been conjectured that capillary blood result variation may be influenced by specimen collection factors that are not yet widely assessed [3]. Similar trends with other devices have been reported elsewhere [3,13], with capillary blood results generally in line with venous blood results. The use of centrifugation prior to the transportation of blood specimens has also been shown to improve performance on similar comparative studies [13] and is a possible suggestion for future analysis, especially for measuring potassium, which is also known to be affected by haemolysis [29]. It is also important to note the potential pitfalls in statistical analyses for measures of agreement [30]. Concordance correlation, Bland–Altman and percentage similarity should not be used as standalone measures of agreement. In our study, although LDH, creatinine and potassium reported moderate to poor concordance correlation, the absolute differences (Bland–Altman and percentage similarity) did not highlight clinical relevance. The sequence and specimen collection time may also be useful variables for evaluating trends in device performance.

Our study was limited by the small number of Onflow devices available for evaluation from the manufacturer. Further studies are suggested in applying the Onflow method to patient cohorts (those anticipated with analyte test results beyond the “normal” range) that could also include younger patients. Expanding the use of alternative blood collection tubes, such as K3EDTA or other tube additives, would also expand testing to high-volume tests in the region, such as HIV viral load, for monitoring response to antiretroviral therapy. However, each new additive type would require formal evaluation to ensure acceptable performance with capillary whole blood. Patient-centric specimen collection with such devices has been suggested as plausible [1] and even feasible, as with the Tasso device, the TAP^®^ devices or finger stick collection into Microtainers [4,8,9,11,17] and could be suggested for the Onflow device. The advantage of the Onflow, TAP^®^ and Tasso over finger stick capillary blood collection for self-sampling is that the specimen is drawn using a vacuum, and thus, potential interference by tissue fluid through “milking of the finger” can be avoided [3].

## 5. Conclusions

Overall, the Onflow device shows promise as an alternative blood specimen collection method. The analytes measured generated results within acceptable limits and users and participants found the Onflow device user-friendly. The findings of this study set the foundation for further clinical studies in a larger cohort and amongst participants with expected abnormal chemistries, as well as for studies where patients could self-collect blood, therefore reducing times spent in clinics and improving clinic workflows.

## Figures and Tables

**Table 1:** Device usability and acceptability questions.

Question	Answer Options
Participant Questions (answered after collection for each specimen)	
Rate pain of blood draw procedure	1 (No pain)–10 (Most painful event)
How likely is it that you would use this method again?	1 (Extremely unlikely)–5 (Extremely likely)
Rate ease of use of blood collection procedure.	1 (Difficult)–5 (Easy)
HCP questions	
Onflow questions (answered before collection)	
Was the sterile barrier patch intact?	Yes/No
Was the blade shielded before use?	Yes/No
Was the tube in good condition (i.e., without cracks, marks or otherwise compromised)?	Yes/No
Onflow questions (answered after collection)	
Was the device able to be activated with one hand?	Yes/No
Did you hear the device activate (click)?	Yes/No
How long did the collection take?	mm.ss
Ease of use (answered after collection)	
Rate difficulty in closing the tube.	1 (Difficult)–5 (Easy)
Rate the effect of the device on the skin surface after collection. *Adapted from FDA Draft Guidance (2018)* [20]	0–No bruising or redness 1–Slight redness, but no bruising 2–Slight redness and slight bruising 3–Moderate redness, but no bruising 4–Severe redness and/or bruising
Rate fit of the device on the subject’s arm.	1 (Poor)–10 (Good)
Rate IFU clarity and ease of model identification.	1 (Difficult)–10 (Easy)
Rate ease of use of the device.	1 (Difficult)–10 (Easy and intuitive)

FDA: Food and Drug Administration (United States of America); HCP: health care practitioner; IFU: instructions for use; mm.ss: minutes and seconds.

**Table 2:** Participant demographics.

Category	Total
Total number of participants	100 ^1^
Gender—n	99
Male—n (%)	46 (46.5)
Female—n (%)	53 (53.5)
Age (years)—mean (range)	34.9 (19, 54)
Male (years)—mean (range)	33.5 (19, 51)
Female (years)—mean (range)	36.2 (22, 54)
Height (cm)—mean (range)	166.2 (146.0, 186.0)
Male (cm)—mean (range)	172.6 (159.0, 186.0
Female (cm)—mean (range)	160.3 (146.0, 176.0)
Weight (kg)—mean (range)	78.4 (50.1, 139.1)
Male (kg)—mean (range)	77.3 (50.1, 110.7)
Female (kg)—mean (range)	79.4 (55.8, 139.1)
BMI (kg/m^2^)—mean (range)	28.6 (18.9, 46.6)
Male (kg/m^2^)—mean (range)	26.0 (18.9, 37.0)
Female (kg/m^2^)—mean (range)	30.9 (19.1, 46.6)
Race—n (%)	99 (100)
Black African—n (%)	91 (91.91)
Asian or Indian—n (%)	3 (3.03)
Coloured ^2^—n (%)	3 (3.03)
White—n (%)	2 (2.02)
Medications—n (%)	8 (8.08)

^1^ One participant (male, 43 years old and of Asian/Indian origin, BMI = 27 kg/m^2^) was excluded from the study as he experienced a mild adverse event (profuse sweating and anxiety, but with normal vital signs). The attending medical staff did not consider this to be related to the use of the Onflow device, but rather to anxiety regarding potential pain and the use of an unknown device. The patient recovered within 10 min and reported no pain on the Onflow collection site and no bruising. It would be to the benefit of the supplier to further emphasize the painless nature of the blood collection.”. ^2^ Coloured is a recognised population group in South Africa. %: percentage; BMI: body mass index; cm: centimeters; kg: kilograms; kg/m^2^: kilograms per square meter; n: number.

**Table 3:** Post-blood draw characteristics.

Category	Result		
Specimen Type (n = 99 of each)	Venepuncture	Loop One 1	Loop One 2
**Skin Appearance—n (%)**			
Pre-draw: normal appearance, no apparent abnormalities)	99 (100.00)	99 (100.00)	99 (100.00)
Post-draw dermal assessment			
0 (no bruising or redness)	Not assessed	94 (94.95)	94 (94.95)
1 (slight redness, but no bruising)		5 (5.05)	5 (5.05)
**Specimen collection data**			
Left arm—n (%)	48 (48.48)	84 (84.85)	15 (15.15)
Right arm—n (%)	51 (51.51)	15 (15.15)	84 (84.85)
No repeats—n (%)	98 (98.99)	91 (91.92)	90 (90.91)
One repeat required—n (%)	0 (0.00)	7 (7.07)	9 (9.09)
Two repeats required—n (%)	1 (1.01)	1 (1.01)	0 (0.00)
Successful collection with acceptable blood volume—n (%)	99 (100.00)	96 (96.97)	99 (100.00)
Time for collection (mm.ss)—median, mean (range)	Not assessed	05.59, 06.25 (02.05, 11.25)	06.53, 06.42 (02:08, 12:47)
Blood weight ^1^ (g)—mean (range)	Not assessed	1.203 (0.218, 1.748)	1.171 (0.225, 1.793)
Estimated blood volume ^1^ (mL)—mean (range)	Not assessed	1.135 (0.206, 1.649)	1.187 (0.212, 1.692)

^1^ Blood weight calculated by subtracting the mean tube weight from the weight measured in the laboratory. Blood volume is calculated by dividing the blood weight by the density of blood. %: percentage; g: grams; mL: millilitres; mm.ss: minutes and seconds; n: number.

**Table 4:** Analyte method comparison of Onflow and venous blood collection for five analytes that were successfully reported. Concordance correlation, Bland–Altman and percentage similarity analyses were performed, and their respective outputs included with concordance correlation and Bland–Altman difference plots for visual comparison.

Alanine transaminase (n = 186)	Venous mean (range): 25.03 U/L (6, 100) Onflow mean(range): (24.54 U/L (5, 95)
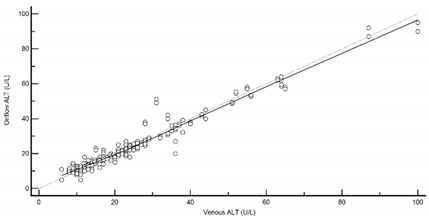 P_c_ = 0.976 (0.968 to 0.982), substantial agreement ρ = 0.977 c_b_= 0.999	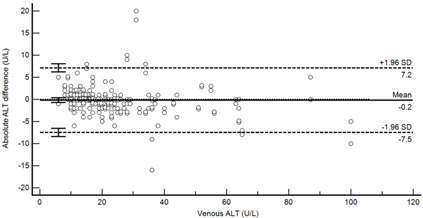 Mean bias (95%CI) = −0.161 U/L (−0.701 to 0.378) Mean %Similarity = 100.7% %Similarity SD = 9.0%, %Similarity CV = 9.0%
Aspartate transaminase (n = 188)	Venous mean (range): 24.79 U/L (11, 70) Onflow mean (range): 24.63 U/L (7, 68)
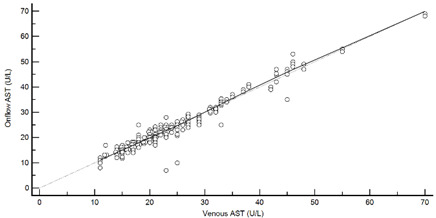 P_c_ = 0.970 (0.960 to 0.977), substantial agreement ρ = 0.971 c_b_ = 0.999	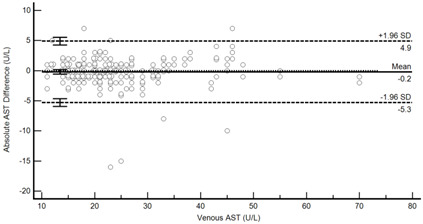 Mean bias (95%CI) = −0.197 U/L (−0.571, 0.177) Mean %Similarity = 99.7% %Similarity SD = 5.5%, %Similarity CV = 5.5%
Creatinine (n = 188)	Venous mean (range): 69.8 µmol/L (39, 111) Onflow mean (range): 64.1 µmol/L (36, 107)
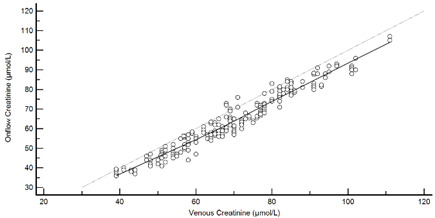 P_c_ = 0.912 (0.889 to 0.930), Moderate agreement ρ = 0.972 c_b_ = 0.938	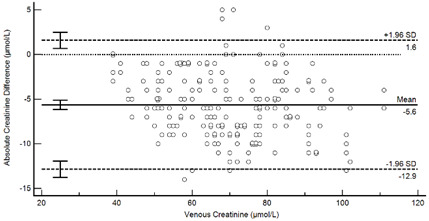 Mean bias (95%CI) = −5.633 µmol/L (−6.163, −5.103) Mean %Similarity = 96.0% %Similarity SD = 2.7%, %Similarity CV = 2.8%
Lactate dehydrogenase (n = 188)	Venous mean (range): 210 U/L (137, 330) Onflow mean (range): 226 U/L (139, 345)
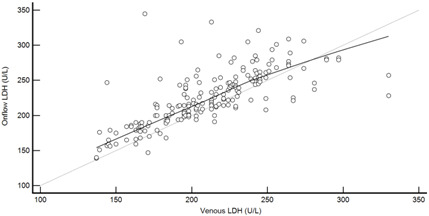 P_c_ = 0.673 (0.593 to 0.740), Poor agreement ρ = 0.724 c_b_ =0.931	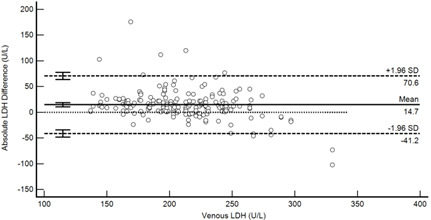 Mean bias (95%CI) = 14.707 U/L (10.603, 18.812) Mean %Similarity = 104.0% %Similarity SD = 7.0%, %Similarity CV = 6.7%
Potassium (n = 188)	Venous mean (range): 4.5 mmol/L (3.7, 5.5) Onflow mean (range): 4.6 mmol/L (3.4, 5.8)
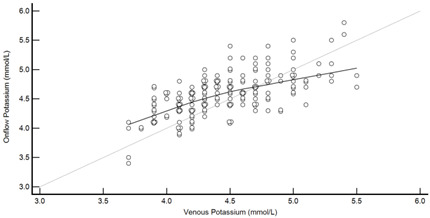 P_c_ =0.617 (0.526 to 0.795), Poor agreement ρ = 0.653 c_b_ = 0.946	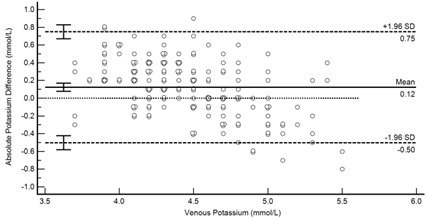 Mean bias (95%CI) = 0.124 mmol/L (0.078, 0.170) Mean %Similarity = 101.6% %Similarity SD = 3.6%, %Similarity CV = 3.6%

%Similarity: percentage similarity; 95%CI: 95 percent confidence interval; c_b_: bias correction factor; CV: coefficient of variation; µmol/L: micromoles per litre; mmol/L: millimoles per litre; n: number; P_c_: concordance correlation coefficient; ρ: Pearson precision; U/L: units per litre; SD: standard deviation.

## Data Availability

The data presented in this study are available on request from the contact author. The data is not shared due to restrictions governed by the South African Act 4 of 2013 Protection of Personal Information Act (https://www.gov.za/sites/default/files/gcis_document/201409/3706726-11act4of2013protectionofpersonalinforcorrect.pdf, accessed 11 May 2023).
